# Psychological Therapies in Patients with Irritable Bowel Syndrome: A Systematic Review and Meta-Analysis of Randomized Controlled Trials

**DOI:** 10.1155/2015/549308

**Published:** 2015-01-31

**Authors:** Osama Altayar, Varun Sharma, Larry J. Prokop, Amit Sood, Mohammad Hassan Murad

**Affiliations:** ^1^Department of Internal Medicine, Allegheny General Hospital-Western Pennsylvania Hospital Medical Education Consortium, Pittsburgh, PA, USA; ^2^Division of General Internal Medicine, Mayo Clinic, Rochester, MN, USA; ^3^Center for the Science of Health Care Delivery, Mayo Clinic, Rochester, MN, USA

## Abstract

*Background*. Irritable bowel syndrome (IBS) is a poorly understood disease with few effective treatments. Psychosocial factors are believed to contribute to the pathogenesis of IBS. *Objective*. To evaluate the evidence for psychological therapies in IBS treatment. *Methods*. We searched six medical databases through February 6, 2014, for randomized controlled trials (RCTs) of psychological therapies for the treatment of IBS. Two independent reviewers identified the RCTs, extracted the data, and assessed trial quality. We used the random-effect model to pool standardized mean difference (SMD) and 95% confidence interval (CI) across trials. *Results*. 15 RCTs that mostly evaluated cognitive behavioral therapy were included. Psychological therapies were associated with improvement in IBS symptoms severity scales (SMD −0.618; 95% CI: −0.853 to −0.383), IBS-Quality of Life (SMD 0.604; 95% CI: 0.440 to 0.768), and abdominal pain (SMD −0.282; 95% CI: −0.562 to −0.001). No statistically significant effect was observed on diarrhea or constipation. *Limitations*. The trials were at increased risk of bias and the overall sample size was small leading to imprecision. *Conclusion*. Psychological therapies may improve the quality of life and symptom severity in IBS. The effect size noted is moderate to large and is clinically meaningful.

## 1. Introduction

Irritable bowel syndrome (IBS) is a complex and widespread functional bowel disorder (10–20% worldwide prevalence [1]) that is not well understood. IBS typically presents as persistent diarrhea and/or constipation that is accompanied by abdominal discomfort. The symptoms as well as the underlying etiologies of IBS can vary considerably from patient to patient. Some of these causes may include diet, genetics, altered intestinal environment, and dysregulation of the enteric nervous system function. Multiple treatments targeting these possible causes have been used for several decades but have largely only been demonstrated to temporarily treat symptoms.

Recently, acknowledgment of the role of stress and psychosocial factors in some cases has led to the examination of psychological therapies targeting these factors in the treatment of IBS. In the past few decades, research has uncovered an extensive bidirectional communication network between the brain and the gut termed the brain-gut axis [2]. This provides a pathophysiologic basis for the potential therapeutic effects of psychological therapies on gut function. This has been further supported by several, small, randomized controlled trials demonstrating the preliminary efficacy of psychological therapies on IBS symptoms [3–7]. Hypnotherapy has been suggested to treat abdominal pain, improve quality of life, and reduce anxiety and depression in IBS without any side effects [8–10]. These effects persisted for several years, although definitive conclusions will require larger, higher quality studies. Cognitive behavioral therapies (CBT) and mind-body therapies (MBT) have also been studied in IBS with some studies showing preliminary efficacy [11–13]. Psychological therapies are potentially efficacious in treating IBS symptoms in many patients, and unlike many pharmaceutical treatments, they have minimal side effects and can be cost-effective [14–17].

To examine if psychological therapies merit incorporation in the clinical treatment of IBS, we conducted this systematic review and meta-analysis of published randomized controlled trials. To our knowledge, no existing systematic reviews with meta-analysis have addressed this question.

## 2. Methods

Investigators developed a protocol in advance to specify eligibility criteria, outcomes of interest, and analysis methods. The methodology and reporting of this systematic review comply with the Preferred Reporting Items for Systematic Reviews and Meta-Analyses (PRISMA statement) [18].

### 2.1. Eligibility Criteria

We included randomized controlled trials that enrolled patients of unspecified gender and aged at least 18 years. Subjects of the included trials were diagnosed with irritable bowel syndrome (IBS) based on one of the following criteria: Latimer criteria, Manning criteria, Kruis criteria, Rome I criteria, Rome II criteria, Rome III criteria, or clinician defined diagnosis [12, 15, 17, 19–38]. We included trials that evaluated the efficacy of psychological interventions, including cognitive-behavioral therapies, mind-body therapies, and other psychological interventions, compared to no intervention, waiting list, placebo, diet, herbal treatment, or symptomatic management. Only trials that evaluated the efficacy of psychological interventions using composite IBS symptoms severity scales, individual IBS symptoms severity scales, or quality of life scales were included.

Nonrandomized comparative studies and single arm studies were not included. We excluded trials that evaluated hypnotherapy because multiple systematic reviews have already summarized this evidence [8–10]. We also excluded non-English references.

### 2.2. Search Methods

A comprehensive search of several databases from 1966 to February 6, 2014, any language, was conducted. The databases included Ovid Medline In-Process & Other Non-Indexed Citations, Ovid MEDLINE, Ovid EMBASE, Ovid PsycINFO, Ovid Cochrane Central Register of Controlled Trials, Ovid Cochrane Database of Systematic Reviews, and Scopus. The search strategy was designed and conducted by an experienced librarian with input from the study's principal investigator. Controlled vocabulary supplemented with keywords was used to search for comparative studies of psychological and mind-body interventions for irritable bowel syndrome. The actual strategy is included in the Appendix.

### 2.3. Study Selection

Two independent blinded reviewers (OA and VS) assessed the eligibility of the candidate references for inclusion by screening titles and abstracts initially. The full-text publications were then retrieved and their eligibility was assessed. Disagreement between the two reviewers was resolved by meeting and establishing consensus. Interreviewer agreement was measured by using the Kappa statistic.

### 2.4. Data Collection Process

Data were abstracted from each study using a standardized and piloted Microsoft Excel spreadsheet-based extraction form. Two independent blinded reviewers (OA and VS) did the abstraction in duplicate and disagreements were resolved by meeting and establishing consensus. The following data were abstracted: description of enrolled patients (inclusion criteria, age, gender, race, and previous treatment), description of received interventions and control, follow-up monitoring, and measures of outcome.

### 2.5. Outcomes of Interest

The primary outcomes were the composite IBS symptoms severity scales and quality of life. Other outcomes were diarrhea, constipation, and abdominal pain.

### 2.6. Assessment of the Risk of Bias

Two reviewers (OA and VS) evaluated the methodological quality of the included trials. To evaluate randomized controlled trials, we used the Cochrane Risk of Bias tool [39]. We evaluated the adequacy of randomization, allocation concealment, blinding (patients, providers, data collectors, and outcome assessors), baseline imbalance, and extent of loss to follow-up. We also extracted the funding source.

### 2.7. Statistical Analysis

Because the outcomes of interest were evaluated in the included trials using different scales, we estimated the standardized difference in means (SMD) to measure the difference between the intervention and control groups. SMD calculation involves standardizing the effect and expressing it in standard deviation units, to allow pooling it across trials. For each trial, we calculated the change in the studied scales before and after the intervention and compared it to the change in the control group. Then DerSimonian and Laird random-effects model was used to pool SMD across trials [40].

Inconsistency across the trials was assessed using the *I* ^2^ static and Cochran's *Q* test. *I* ^2^ value more than 50% was considered indicative of substantial heterogeneity that is due to real differences in protocols, trial populations, interventions, and/or outcomes. Also, Cochran's *Q* test *P* value less than 0.05 indicates that the heterogeneity is beyond chance or random error [41]. We planned to conduct formal tests to assess potential publication bias using visual inspection of funnel plots and Egger's regression asymmetry tests but this was not possible due to the small number of trials [42].

We planned to explore possible causes of heterogeneity by conducting subgroup analyses comparing the effect size between trials that evaluated CBT versus other forms of psychotherapy, trials in which patients received prior treatment versus those who did not, trials with high risk of bias versus low risk of bias, trials in which placebo or sham therapy was used in the control arm, and trials in which the control arm received pharmacological treatment versus those that did not. Interaction test between subgroups was done as suggested by Altman and Bland [43].

Statistical analyses were conducted using Comprehensive Meta-Analysis Version 2.2 [44].

## 3. Results

### 3.1. Study Selection

**Figure 1:**
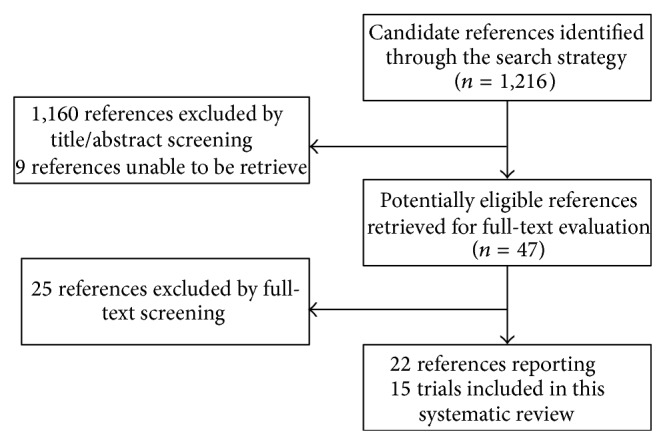
Summary of selection process.

A total of 1,216 references were identified through the search strategy. Screening of titles and abstracts excluded 1,160 references (Figure 1). Two reviewers performed the initial screening and had an interreviewer agreement of Kappa of 0.84. Reviewing the retrieved full texts by the two reviewers excluded 25 publications and the interreviewer agreement about study eligibility, Kappa static, was 0.87. The remaining 22 publications included 15 trials.

### 3.2. Study Characteristics

The 15 included RCTs enrolled 1,352 patients. The follow-up period ranged from 10 weeks to 15 months. The criteria for the included patients, baseline characteristics of the included patients, and the interventions they received are detailed in Table 2.

Greene used the Latimer criteria [38] to diagnose IBS while Fernandez used the Manning criteria [45]. Seven of the fifteen trials used Rome I criteria [46] and five trials used the Rome II criteria [47]. Corney et al. used author specified criteria [22].

The included trials evaluated multiple psychological interventions: cognitive-behavioral therapies, psychoeducational courses, mind-body therapy, psychodynamic interpersonal therapy, and contingency management [12, 15, 17, 19–37].

These interventions were compared to treatment as usual and routine clinical care, providing reading material, attention control, symptom and stressful event monitoring, waiting lists, support groups, standard medical therapy, or placebo [12, 15, 17, 19–37].

The mean age of the included patients ranged from 34 to 50 years. The majority of patients (1,067/1,352) were females. Five of the included trials reported previous treatment. Four of them asked the patients to continue the current treatment and the fifth study had the patients go through a 2-week washout period [12, 15, 17, 19–37].

### 3.3. Risk of Bias within Trials

All the included trials were randomized controlled trials. Assessment of risk of bias for each of the included trials is summarized in Table 1. Nine of the fifteen trials had high risk of bias. All these nine trials did not provide details about allocation concealment. Seven of them did not report the randomization method. For the remaining two trials, one of them did not report the presence of baseline imbalances and blinding, and the other one had inadequate randomization.

Five of the fifteen included trials had moderate risk of bias. Two of the five had high loss to follow-up rate (44.5% and 50.5%). The remaining three trials did not report whether allocation was concealed or not. Only one of the fifteen trials was found to have a low risk of bias.

Thirteen of the included trials were funded by a not-for-profit organization. One study did not disclose funding source and one study was funded partially by a pharmaceutical company.

### 3.4. Meta-Analysis

**Figure 2:**
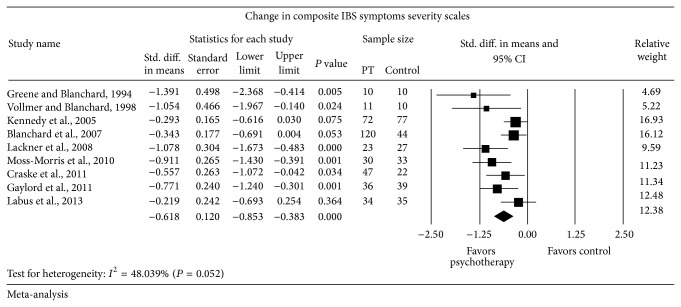
Forest plot for composite IBS symptoms severity scales in RCTs of psychotherapy (PT) versus control.

Nine of the included trials reported change in composite IBS symptoms severity scales. Four of these trials used the Irritable Bowel Syndrome-Severity Scoring System (IBS-SSS) used by Francis et al. [48], three used the Composite Primary Symptoms Reduction (CPSR) score used by Blanchard and Schwarz [49], one used the composite Bowel Symptom Severity (BSS) score used by Spiegel et al. [50], and one used a Global GI Symptoms Severity Score [30]. Random-effects meta-analysis (Figure 2) showed a statistically significant change in composite IBS severity scales in patients who received psychological therapy (*n* = 383) compared to patients receiving control (*n* = 297) with SMD = −0.618 SD favoring psychological therapy (95% CI from −0.853 to −0.383). Moderate heterogeneity was observed (*I* ^2^ = 48.04% and *P* = 0.052).

**Figure 3:**
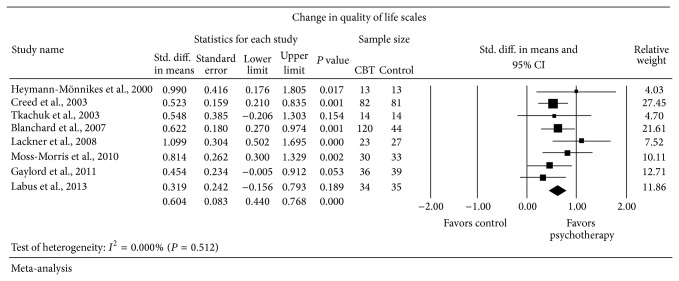
Forest plot for quality of life scales in RCTs of psychotherapy (PT) versus control.

Eight of the included trials reported change in quality of life scales. Three of the included trials used the Irritable Bowel Syndrome-Quality of Life (IBS-QOL) scale used by Drossman et al. [51, 52], two trials used the Physical Component Score of the Short Form 36 Health Survey [53], one study used the Irritable Bowel Syndrome-Quality of Life scale used by Hahn et al. [54], one study used the GI Quality-of-Life Index used by Eypasch et al. [55], and one study used the Work and Social Adjustment Scale used by Mundt et al. [56]. Random-effects meta-analysis (Figure 3) showed a statistically significant change in quality of life scales in patients receiving psychological therapy (*n* = 352) compared to patients receiving control (*n* = 286) with SMD = 0.604 SD favoring psychological therapy (95% CI from 0.440 to 0.768). No evidence of heterogeneity was observed (*I* ^2^ = 0.000% and *P* = 0.512).

**Figure 4:**
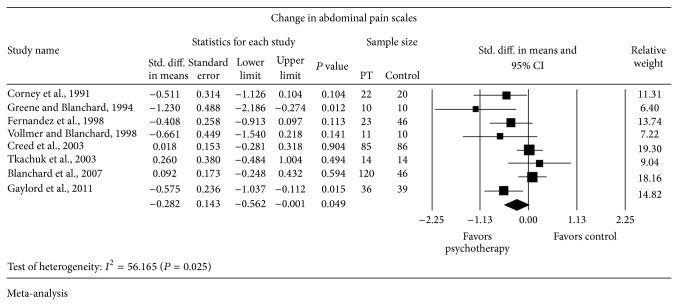
Forest plot for abdominal pain scales in RCTs of psychotherapy (PT) versus control.

Eight trials reported change in abdominal pain scales (Figure 4). There was a statistically significant change favoring psychological therapy (*n* = 321) compared to control (*n* = 271) on random-effects meta-analysis with SMD = −0.282 SD (95% CI from −0.562 to −0.001). There was strong evidence of heterogeneity (*I* ^2^ = 56.16% and *P* = 0.025).

**Figure 5:**
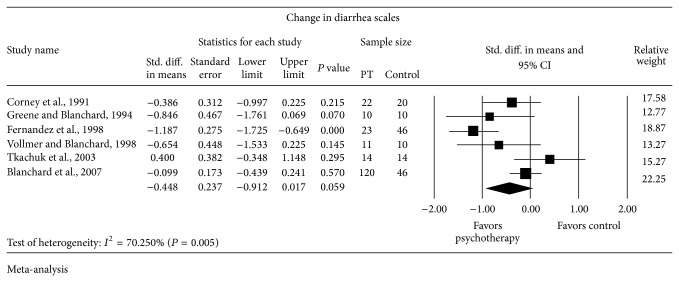
Forest plot for diarrhea scales in RCTs of psychotherapy (PT) versus control.

**Figure 6:**
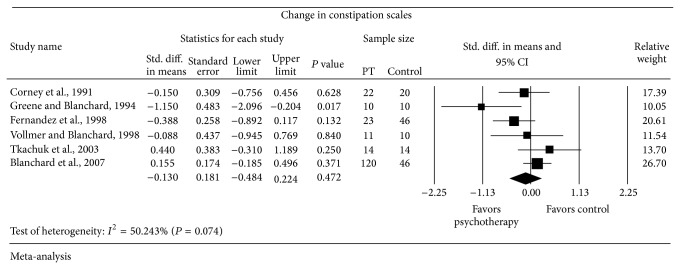
Forest plot for constipation scales in RCTs of psychotherapy (PT) versus control.

Six trials reported changes in diarrhea scales and constipation scales (Figures 5 and 6). Random-effects meta-analyses showed no statistically significant difference in the change of diarrhea scales with SMD = −0.448 SD (95% CI from −0.912 to 0.017) and constipation scales with SMD = −0.130 SD (95% CI from −0.484 to 0.224) between subjects who received psychological therapy (*n* = 200) and who received the control (*n* = 146). Strong evidence of heterogeneity was observed in both meta-analyses of the diarrhea scales (*I* ^2^ = 70.25% and *P* = 0.005) and the constipation scales (*I* ^2^ = 50.24% and *P* = 0.074).

### 3.5. Subgroup Analysis

There was no statistically significant interaction based on the type of treatment (CBT versus other forms of psychotherapy), based on the risk of bias, or based on whether the control arm received pharmacological treatment (*P* > 0.05, Table 3). There were insufficient data to conduct subgroup analyses based on whether a placebo was used in the control arm or based on whether patients received prior treatment.

## 4. Discussion

We conducted a systematic review and meta-analysis of randomized controlled trial evaluating the effects of psychological therapies such as cognitive-behavioral therapy (CBT) and mind-body therapy (MBT) on IBS patients' symptoms and quality of life.

### 4.1. Main Findings

Our search identified 15 trials evaluating a psychological therapy on a sample of IBS patients. We excluded trials of hypnotherapy, as three reviews have already examined its effect on IBS. Outcomes were evaluated using validated scoring scales. The body of evidence varied from small in measures of diarrhea (346 subjects) and constipation (346 subjects) to moderate for the composite IBS symptom severity scales (680 patients). The conducted meta-analyses demonstrated a statistically significant effect of psychological therapies on IBS-Quality of Life and composite IBS symptom severity scales with minimal heterogeneity. In contrast, psychological therapies had no statistically significant effect on diarrhea and constipation with evidence of heterogeneity observed. Psychological therapies had a statistically significant effect on abdominal pain; however, this inference was limited by heterogeneity. It should be noted that the studies assessing these secondary outcomes might not have been adequately powered to evaluate changes in symptoms, as they were either secondary outcomes or part of a composite scale. The pooled effect sizes ranged from 0.13 SD to 0.62 SD.

The standardized difference in the means was used to express the effect size for each outcome in standard deviation unit as each outcome was assessed using different scales. According to Cohen, a difference of 0.2 SD is considered a small difference, 0.5 SD is considered moderate, and 0.8 SD is considered large [57]. Norman found that a 0.5 SD was the minimally clinically important difference (MCID) for changes in most of health-related quality of life for chronic diseases [58]. Studies were done to validate and define the MCID for the IBS-Quality of Life scale [51, 52]. The MCID was found to be 10 to 14 points which is equal to 0.5 to 0.7 standard deviation units [59]. In this meta-analysis, the psychological therapy increased the quality of life scales by 0.604 SD units (95% CI from 0.440 to 0.768) compared to control. Using the above MCID this translates to a clinically meaningful improvement in quality of life.

When Francis et al. described the IBS-Severity Scoring System (IBS-SSS), they found that a change of 50 points was sufficient to detect clinical improvement [48]. This is equal to 0.6 SD in most of the trials that used the IBS-SSS in this meta-analysis [12, 28, 33, 34]. The psychological interventions decreased the symptom severity scales by 0.618 SD (95% CI from −0.853 to −0.383) compared to control in the current meta-analysis. This translates to a clinically meaningful change consistent with improvement in the IBS symptoms severity scales.

The meta-analysis of the abdominal pain showed a statistically significant difference. We were not able to find a study that established the MCID for abdominal pain scales in patients with IBS. The psychological therapies decreased the abdominal pain scales by 0.282 SD (95% CI from −0.562 to −0.001) and by applying Cohen's assumption [57] above this translates to a small difference. Also, this statistically significant difference was limited by the presence of heterogeneity.

The quality of evidence according to the GRADE [60] approach for the primary outcomes was low and moderate for IBS symptom severity scale and IBS-Quality of Life, respectively, rated down for high and moderate risk of bias. The quality of evidence for secondary outcomes was very low, with downratings due to the overall high risk of bias, serious imprecision, and serious inconsistency in all of them.

### 4.2. Strengths and Limitations

By its nature IBS is a poorly understood, heterogeneous disease with varied clinical presentations and underlying etiologies. Despite this fact, all the trials evaluated used very similar protocols and criteria for defining their sample population of IBS patients, even if the criteria for IBS have evolved over the three decades from which these trials were conducted. Further strengths of this systematic review relate to the reviewers' measures taken to control bias. Some of these measures included study screening, quality evaluation, and data extraction in duplicate. The search strategy was comprehensive, extracting trials from multiple databases. To our knowledge, this is the first systematic review with accompanying meta-analysis to quantitatively measure the effects of psychological therapies, other than hypnotherapy, on gastrointestinal symptoms in patients with IBS.

There are many limitations in this review. The trials evaluated had many methodological limitations and were generally small. Although specific details on frequency and duration of the psychological interventions were given in the trials, the efficacy of psychological therapies such as cognitive-behavioral therapy can range considerably depending on the training and experience of the therapist. The therapy is also often modified in the presence of psychological comorbidity, which is common in IBS patients. This can result in variability in the therapy given in the different trials. There was variability in the follow-up periods as well. Publication bias is likely in a field in which evidence consists of trials with small size. Further, the efficacy of CBT or MBT could be driven primarily by improvement in general feeling of well-being and lower stress, and not specific improvement in IBS pathophysiology.

Another limitation pertains to the heterogeneity of IBS itself. IBS is now recognized as including three main subtypes: IBS-diarrhea predominant, IBS-constipation predominant, and IBS-mixed type. Many patients also develop IBS as sequelae to gastrointestinal infection or what is termed postinfectious IBS. These subtypes may have varying etiologies underlying their pathophysiology. By failing to stratify results by these subtypes, we are unable to know if there would be differences in efficacy of psychological therapies between subtypes. This would have important practical implications for the clinical incorporation of psychological therapies in the treatment of IBS. In terms of the analysis, heterogeneity that remained unexplained despite subgroup analyses lowers the confidence in the meta-analytic estimates.

## 5. Conclusion

Psychological therapies such as cognitive-behavioral therapy and mind-body therapy may help to improve gastrointestinal symptoms and quality of life in IBS patients. Although statistical significance was found in IBS measures of quality of life and symptom severity, these results should be interpreted with caution as trials were generally of low quality. Future trials will require larger sample sizes, longer follow-up periods, and higher quality methodology to provide a definitive recommendation on the incorporation of psychological therapies in the treatment of IBS. However, despite these concerns, psychological therapies appear to be a safe intervention and could be a practical option for patients who fail standard medical therapy.

## Figures and Tables

**Table 1:** Risk of bias assessment in the included trials.

Author, year (reference)	Randomization method	Allocation concealment	Blinding	Baseline imbalance	Lost to follow-up	Source of funding
Corney et al., 1991 [22]	NR	Unclear	NR	No significant differences	2.38%	NR

Greene and Blanchard, 1994 [25]	NR	NR	NR	Matched subjects (GI symptoms, axis I psychiatric diagnosis, sex, duration of IBS, and age)	7.14%	NFP

Fernandez et al., 1998 [24]	NR	NR	NR	No significant difference	29.54%	NFP

Vollmer and Blanchard, 1998 [36]	NR	NR	NR	Matched subjects (DSM-IV axis I psychiatric diagnosis, IBS subtypes, age, and duration of IBS symptoms)	NR	NFP

Heymann-M$\ddot{\text{o}}$nnikes et al., 2000 [26]	Unclear	Unclear	NR	NR	7.7%	NFP

Boyce et al., 2003 [20]	Adequate Random number generator	Sealed opaque envelopes	RCC provider and data collector	Participants were similar	50.48%	NFP

Creed et al., 2003; Hyphantis et al., 2009 [15, 27]	Adequate Computer-generated random numbers Stratified by hospital and by pain severity	Unclear	Assessors	No difference in demographic or diagnostic variables	13.62%	NFP

Tkachuk et al., 2003 [35]	NR	NR	NR	Matched subjects (DSM axis I disorder, IBS subtype, IBS symptom duration, age, and gender)	NR	NFP

McCrone et al., 2008; Kennedy et al., 2005;Kennedy et al., 2006 [17, 28, 29]	Adequate Random number tables	Unclear	NR	No significant difference	11.41%	NFP

Blanchard et al., 2007;Lackner et al., 2007 [19, 32]	Unclear	Not reported	Not reported	No between-group differences at baseline	10.48%	NFP

Lackner et al., 2010;Lackner et al., 2008 [31, 33]	Adequate Computer-generated random numbers	Unclear	NR	NR	16%	NFP

Chilcot and Moss-Morris, 2013;Moss-Morris et al., 2010 [21, 34]	Adequate Computer-generated random numbers	Sealed opaque envelops	Assessors	Intervention group (CBT-TAU) reported higher HADS anxiety scale	1.56%	NFP

Craske et al., 2011; Wolitzky-Taylor et al., 2012 [23, 37]	Adequate Random number generator	Opaque envelopes	Assessors	No statistically significant difference	44.54%	NFP

Gaylord et al., 2011 [12]	Adequate Computer-generated random numbers	NR	Patients, data collectors, and managers	No statistically significant difference	12%	NFP

Labus et al., 2013 [30]	Inadequate Quasi-randomized	No	No	Intervention group (psychoeducation group) reported lower GI symptom severity score and higher HADS anxiety score and level of education	NR	Includes FPO GlaxoSmithKline

NR: not reported; GI: gastrointestinal; IBS: irritable bowel syndrome; NFP: not-for-profit organization; DSM: Diagnostic and Statistical Manual of Mental Disorders; RCC: routine clinical care; CBT: cognitive behavioral therapy; TAU: treatment as usual; HADS: Hospital Anxiety and Depression Scale; FPO: for-profit organization.

**Table 2:** Baseline characteristics and description of interventions.

Author, year, country, and objective	Inclusion/exclusion criteria	Number of patients (*N*) and baseline characteristics	Length of follow-up	Interventions; number of patients (number of patients lost to follow-up) Description of intervention
**Corney et al., 1991 [22]** **Country**: UK **Objective**: to establish if stress management is superior to medical management in IBS and if psychosocial factors predict therapeutic response.	**Inclusion**: abdominal pain that fits no other disease pattern and/or nonbloody diarrhea and/or constipation with discomfort; 6 months of symptoms; normal sigmoidoscopy and rectal biopsy; and altered bowel habits. **Exclusion**: presence of psychological disorders as rated by CIS (>13).	**N**: 42 **Age**: range 19–73. Half were aged under 30. **Gender**: 74% females. **Race**: NR **Previous treatment**: NR	9 months	**BT; 22 (1)** Behavioral therapy. 6–15 weekly one-hour sessions with nurse behavior therapist. Discussed symptoms and impact on life. Pain management techniques. **MT; 20 (0)** Medical treatment. 1–4 outpatient appointments; treatment using explanation, reassurance, medications, antispasmodics, bulk laxatives, and dietary advice.

**Greene and Blanchard, 1994 [25]** **Country**: USA **Objective**: to investigate if a treatment designed to address anxiety related to GI symptoms can be advantageous to IBS patients.	**Inclusion**: Latimer criteria; age 18–70. **Exclusion**: schizophrenia; bipolar disorder; organic mental disorder; or patients involved in cognitive therapy in the last 12 months.	**N**: 20 **Age**: 38.2 (10.9)^*^ **Gender**: 75% females. **Race**: NR **Previous treatment**: NR	10 weeks	**CT; 10 (2)** Cognitive therapy. Ten individual 1-hour sessions: biweekly for two weeks and then weekly for 6 more weeks. Education on IBS and increased subjects' awareness of association of stressors, thoughts, and appraisals of symptoms. **SM; 10 (0) ** Symptom monitoring condition. Monitored GI symptoms with diary for 10 weeks.

**Fernandez et al., 1998 [24]** **Country**: Spain **Objective**: to explore the superiority of behavioral approaches to IBS over other procedures.	**Inclusion**: Manning criteria; endoscopic workup; IBS for more than a year; at least two of the following: wrong ingestion of medicines prescribed by doctor and/or noncompliance, more than one visit not scheduled by gastroenterologist, worker absenteeism or difficulties with carrying out ordinary job tasks, previous psychiatric treatment, and entry into the emergency service without medical indication. **Exclusion**: NR	**N**: 90 **Age**: 44 **Gender**: 66% females. **Race**: NR **Previous treatment**: NR	12 weeks	**CM; 23 (7) ** Contingency management. Aim to show the patient how to practice adaptive behaviors to IBS symptoms and to extinguish maladaptive behaviors in the presence of those symptoms. 10 weekly sessions. **SM; 21 (6) ** Stress management. Aim to provide the patient with effective techniques to mitigate the physiological effects of stress and tension and to modify his/her coping skills. Daily practice for about 20 minutes. 10 weekly sessions. **CG and PCG; 23 and 23 (4 and 6)** Control group (conventional medical treatment) and placebo control group (some imaginative and active visualization of bowel function exercises and the prompting of their own capacity for self-regulation through thought, stimulating their concentration to the utmost. Daily practice at home and 10 weekly sessions of practice with therapist).

**Vollmer and Blanchard, 1998 [36]** **Country**: USA **Objective**: to investigate whether cognitive therapy in a small group setting could provide a cost-effective alternative to individual therapy.	**Inclusion**: Rome I criteria. **Exclusion**: lab findings, physician examination, irritable bowel disease, intestinal parasites, organic pathology, or pregnancy. Diagnosis of serious psychiatric illness.	**N**: 32 **Age**: 43.47 (12.58)^*^ **Gender**: 78% females. **Race**: NR **Previous treatment**: NR	10 weeks	**ICT; 11 (NR)** Individual cognitive therapy. Increasing subjects' awareness of association of stressors, thoughts, and IBS symptoms. Training subjects to identify and modify cognitive appraisals of behaviors. Change depressive life scripts. Weekly 60-minute sessions for 10 weeks. **GCT; 11 (NR) ** Group cognitive therapy. Same as above but in weekly 90-minute group sessions for 10 weeks. **WC; 10 (NR) ** Waitlist control. 8-week symptom monitoring.

**Heymann-M** $\ddot{o}$ **nnikes et al., 2000 [26]** **Country**: Germany **Objective**: to investigate if behavioral therapy with medical treatment is more effective than medical treatment alone in a tertiary GI referral center.	**Inclusion**: Rome I criteria and medical assessment. **Exclusion**: Mental disorders as detected by screening tests.	**N**: 26 **Age**: 37.8 (14.57)^*^ **Gender**: 81% females. **Race**: NR **Previous treatment**: NR	3 months	**SMBT; 13 (1) ** Standardized multicomponent behavioral treatment. 10 sessions, 60 minutes each over 10 weeks with clinical psychologists in pilot tested behavioral program adapted for IBS. Included: information; shaping of a plausible illness model; progressive muscle relaxation; cognitive coping strategies; problem-solving; assertiveness; and social skills training. **SMT; 13 (1) ** Standard medical therapy. Supportive physician-patient relationship and symptom oriented pharmacotherapy. Met for 30–45 minutes every 2 weeks with GI doctor.

**Boyce et al., 2003 [20]** **Country**: Australia **Objective**: to compare the effects of CBT with relaxation versus clinical care alone in IBS patients.	**Inclusion**: Rome I criteria; no structural bowel pathology accounting for their symptoms; age ≥ 18; speaking sufficient English. **Exclusion**: Any major medical or psychotic illness; history of alcoholism; current psychological treatment and use of antidepressants or antipsychotics; or current use of medications that could affect bowel function.	**N**: 105 **Age**: 42.3 (11.8)^*^ **Gender**: 81% females. **Race**: NR **Previous treatment**: 50% had previously had treatment for IBS at some point. Two-week washout before randomization for all subjects.	12 months	**CBT + RCC; 35 (17)** Cognitive behavioral therapy with routine clinical care. Psychological assessment followed weekly 1-hour CBT sessions over 8 weeks by clinical psychologist. Manual-based program based on hypochondriasis model and CBT approach used for anxiety with IBS modification. Homework included relaxation skills, restructuring cognition, enhanced coping strategies, and symptom appraisal, in addition to RCC. **RT + RCC; 36 (23)** Relaxation training with routine clinical care. Psychological assessment followed by weekly 30 min face to face instructional sessions for 8 weeks on relaxation strategies. Also completed homework assessments of tension, in addition to RCC. **RCC; 34 (13)** Routine clinical care. Three 15–30 min sessions with a gastroenterologist, including medical management, symptom discussion, and dietary fiber advice.

**Creed et al., 2003; Hyphantis et al., 2009 [15, 27]** **Country**: UK **Objective**: to assess the relationship between change in interpersonal difficulties and change in chronic pain, health status, and psychological state in IBS patients.	**Inclusion**: Rome I criteria; age 18–65; duration of symptoms more than 6 months; failure to respond to usual medical treatment for a minimum of 3 months; severe abdominal pain, more than 59 on a visual analogue scale; no contraindication to either psychotherapy or paroxetine; ability to complete the study questionnaires. **Exclusion**: NR	**N**: 257 **Age**: 39.97 (1.37)^*^ **Gender**: 80% females. **Race**: 98% white, 2% other. **Previous treatment**: NR	15 months	**Psychotherapy; 85 (13) ** Psychodynamic interpersonal therapy. One long (2 hours) and 7 short (45 minutes) individual sessions over 3 months. Patients encouraged to discuss their symptoms in depth; links between symptoms and emotional factors were identified. After the 3 months, patients returned to their general practitioner who then managed care. **SSRI; 86 (13)** Selective serotonin reuptake inhibitor. Paroxetine 20 mg orally once a day for 3 months. After the 3 months, patients returned to their general practitioner for management of care. **TAU; 86 (9)** Treatment as usual. Patients continued with routine management under physician supervision.

**Tkachuk et al., 2003 [35]** **Country**: Canada and USA **Objective**: to compare efficacy of 10 sessions of CBGT with a home-based symptom monitoring with weekly telephone contact treatment on refractory IBS patients.	**Inclusion**: Rome I criteria; negative for inflammatory bowel disease, parasites, organic pathology, and pregnancy. **Exclusion**: severe mental disorders including schizophrenia, bipolar disorder, and severe major depression, current drug or alcohol abuse, or organic mental disorder.	**N**: 28 **Age**: 39.5 (12.5)^*^ **Gender**: 96% females. **Race**: NR **Previous treatment**: patients continued to receive medical treatment as usual but were asked to maintain their typical use patterns for the period of the study.	11 weeks	**CBGT; 14 (NR) ** Cognitive-behavioral group therapy. Two cognitive-behavioral therapists cofacilitated groups of 3–8 IBS patients. Ten 90-minute sessions over 9 weeks (2 in the first week, 1 for the next 8 weeks). Focused on patient education and goals; relaxation training; cognitive therapy; assertion training; relapse prevention strategies. **SMTC; 14 (NR) ** Symptom monitoring with weekly telephone contact. Monitored GI symptoms for 13 weeks. Weekly 15-minute phone contact while the same 9 weeks CBGT took place. Patients encouraged to discuss symptoms.

**McCrone et al., 2008; Kennedy et al., 2005; Kennedy et al., 2006 [17, 28, 29]** **Country**: UK **Objective**: to assess the efficacy of cognitive behavior therapy delivered in primary care for treating IBS.	**Inclusion**: Rome I; age 16–50; moderate to severe IBS symptoms despite 2 weeks of usual treatment and 2 weeks of mebeverine therapy. **Exclusion**: pregnancy or breast feeding; alarm symptoms suggestive of colorectal cancer; history of IBD or celiac disease; abdominal pain relieved by acid inhibiting drugs.	**N**: 149 **Age**: 33.7 (9.15)^*^ **Gender**: 85% females. **Race**: NR **Previous treatment**: NR	14 months	**CBT + mebeverine; 72 (11) ** Cognitive behavioral therapy. Six 50-minute sessions delivered by face-to-face contact with a trained nurse, in addition to mebeverine 270 mg three times a day. **Mebeverine; 77 (6)** Mebeverine 270 mg three times a day.

**Blanchard et al., 2007; Lackner et al., 2007 [19, 32]** **Country**: USA **Objective**: to assess the cost savings of group CBG versus individual as well as the efficacy of both treatments on GI symptoms of IBS.	**Inclusion**: Rome II diagnosis. Age greater than 18. **Exclusion**: organic GI disease; very low baseline diary pain ratings; psychotic disorder; severe major depression with moderate to severe suicidal ideation; previous lifetime exposure to CBT.	**N**: 210 **Age**: 49.2 (13.1)^*^ **Gender**: 82% females. **Race**: 95% white, 5% other. **Previous treatment**: NR	5 months	**CBT; 120 (11) ** Cognitive behavioral therapy. 3–6 participant group sessions. Ten weekly 90 min sessions. Explains the role of stress in IBS symptoms; teaches to become observers of cognitions through journaling. Attention directed to cognitive fallacies; attempts to change maladaptive core beliefs and problem-solving. **PS; 46 (6)** Psychoeducational support. 3–6 participants, ten weekly 90 min sessions. Discussions on diet, food sensitivity, diagnostic test education, and physician experiences. Emphasis on sharing views and being supportive. **SSEM; 44 (5) ** Symptom and stressful event monitoring. Participants asked to monitor GI symptoms and stress events daily for 10 weeks. Seen once at midpoint for contact.

**Lackner et al., 2010; Lackner et al., 2008 [31, 33]** **Country**: USA **Objective**: to test the acute treatment effects of self-administered CBT compared to a waitlist control condition in IBS patients.	**Inclusion**: Rome II criteria; age 18–70; willingness to maintain a stable dose of IBS medications during the pretreatment baseline period; minimum 6th grade reading level. **Exclusion**: presence of comorbid organic gastrointestinal disease or mental retardation; concomitant or lifetime participation in psychotherapy featuring cognitive-behavioral techniques; current or past diagnosis of schizophrenia or other psychotic disorders; current diagnosis of unipolar depression with suicidal ideation; and current diagnosis of psychoactive substance abuse.	**N**: 75 **Age**: 46.6 (16.7)^*^ **Gender**: 87% females. **Race**: 95% white, 3% black, 1% Hispanic, and 1% Asian. **Previous treatment**: NR	3 months	**S-CBT; 23 (7)** Standard CBT. Skills-based training program delivered to patients in 10 weekly, 1-hour sessions with weekly assignments. Six overlapping phases: (1) education of stress and IBS, (2) self-monitoring of stress associated with IBS, (3) muscle relaxation, (4) learning to identify, reevaluate, and change negatively skewed thoughts associated with IBS, (5) changing underlying “core” beliefs (e.g., perfectionism) that fuel threatening cognitions, and (6) formal training in problem-solving to strengthen the ability to cope with realistic stressors associated with IBS. **MC-CBT; 25 (5)** Minimal contact CBT. Covers the same range of procedures featured in S-CBT but relies extensively on self-study materials. Meeting for only four 60-minute clinic visits during the same period. Two 10-minute phone contacts are scheduled at weeks 3 and 7 to troubleshoot any problems. Meetings introduced material. **WLC; 27 (0)** Waiting list control. Subjects were placed on a 10-week delayed treatment waiting list, during which time they engaged in daily self-monitoring of gastrointestinal symptoms.

**Chilcot and Moss-Morris, 2013; Moss-Morris et al., 2010 [21, 34]** **Country**: UK **Objective**: to investigate the efficacy of a CBT-based self-management manual for the treatment of IBS.	**Inclusion**: Rome I modified or Rome II criteria; age 18 to 72; could read and write English; living within geographical proximity to the study center. **Exclusion**: suffered from another medical condition that had potential to affect symptoms; had had bowel surgery; had a current serious mental disorder.	**N**: 64 **Age**: 39.5 (16.8)^*^ **Gender**: 72% females. **Race**: 89% white, 11% other. **Previous treatment**: NR.	8 months	**CBT + TAU; 31 (1) ** Cognitive behavioral therapy and treatment as usual. IBS fact sheet in addition to a comprehensive self-management manual and weekly assignments. Also received a 1-hour face-to-face session with a health psychologist at the beginning of the program and two 1-hour therapy sessions by telephone scheduled midway and towards the end. **TAU; 33 (0) ** Treatment as usual. IBS fact sheet included an explanation of how IBS is diagnosed and reassurance that the complete range of tests had been conducted and that their history indicated no structural causes.

**Craske et al., 2011; Wolitzky-Taylor et al., 2012 [23, 37]** **Country**: USA **Objective**: to evaluate the efficacy of a treatment for IBS that directly targets hypervigilance and hypersensitivity to visceral sensations, modeled on the methods used for the treatment of panic disorder.	**Inclusion**: Rome II criteria. **Exclusion**: presence of another chronic pain condition; major mental illness such as schizophrenia and bipolar disorder and substance abuse; or taking narcotic pain medication.	**N**: 110 **Age**: 39.47 (13.50)^*^ **Gender**: 75% females. **Race**: 73% white, 9% black, 2 Hispanic, 10% Asian, and 6% other. **Previous treatment**: subjects continued their usual care, 8.1% on benzodiazepines and 13.5% on antidepressant medication (SSRIs, SNRIs, or TCAs).	6 months	**IE CBT; 47 (22) ** CBT focused on interoceptive cues. Goal of therapy to reduce anxious and avoidant responses to visceral sensations. Consists of IBS symptoms education; attention training; cognitive therapy against visceral sensations; interoceptive exposure to reduce fear of sensations; in vivo exposure to feared situations with IBS symptoms. 10 sessions over 10 weeks, each session lasting 50 minutes. **SM CBT; 41 (18) ** CBT focused on stress management. Goal of therapy to reduce cognitive and physical stressful reactions to daily life events. Consists of education about IBS symptoms and stress; self-monitoring symptoms; muscle relaxation training; cognitive therapy; in vivo exposure to personally stressful situations unrelated to IBS sensations. 10 sessions over 10 weeks, each session lasting 50 minutes. **AC; 22 (9) ** Attention control. Self-monitoring of IBS symptoms; educational material about IBS; and discussions with therapist. 10 fifty-minute sessions over 10 weeks.

**Gaylord et al., 2011 [12]** **Country**: USA **Objective**: to explore the feasibility and efficacy of a group program of mindfulness training for women with IBS.	**Inclusion**: Rome II criteria; female; age 18–75; ability to understand English; willingness to participate. **Exclusion**: diagnosis of mental illness with psychosis; history or current diagnosis of IBD or GI malignancy; uncontrolled lactose intolerance; celiac disease; history of abdominal trauma or surgery involving gastrointestinal resection; or pregnancy.	**N**: 75 **Age**: 42.73 (19.31)^*^ **Gender**: 100% females. **Race**: 72% white, 17% black, and 11% other. **Previous treatment**: subjects continued with their usual care.	5 months	**MG; 36 (2) ** Mindfulness training group. 8 weekly 2-hour sessions plus one half-day retreat. Mindfulness-based stress and pain management program taught by trained mindfulness instructors. Instruction and assignments related to the body scan, sitting and walking meditation, and mindful yoga. The basic course was adapted to an IBS population. Weekly assignments included readings from provided texts: “Full Catastrophe Living” and “IBS for Dummies.” **SG; 39 (7)** Support group. 8 weekly 2-hour sessions plus one half-day retreat. A social-support group intervention led by master's level social workers to control for expectations of benefit and amount of group contact. Focused on predesignated topics and open group discussions about subjects' experiences and reactions to the topic. Weekly assignments included readings from the provided text: “IBS for Dummies.”

**Labus et al., 2013 [30]** **Country**: USA **Objective**: to investigate the efficacy of a CBT-based self-management manual for the treatment of IBS.	**Inclusion**: Rome II criteria; organic disease was excluded with appropriate testing, and a clinical diagnosis of IBS was made by an experienced gastroenterologist. **Exclusion**: NR	**N**: 69 **Age**: 46.8 (12.6)^*^ **Gender**: 72% females. **Race**: 84% white, 9% black, 3% Hispanic, 1% Asian, and 3% other. **Previous treatment**: subjects were instructed to continue their IBS care.	3 months	**Psychoeducational course; 34 (NR)** A course led by a gastroenterologist (45%) with a therapist (55%) and consisting of 5 consecutive weekly 2-hour sessions in a group setting with 5–8 participants per group. Participants were also given reading and practical homework assignments related to the topics covered in each session. The course consisted of an educational component, psychological component, relaxation training, and homework assignments, in addition to chapters from “IBS and the Mind-Body Brain-Gut connection.” **Waitlist; 35 (NR) ** Chapters from “IBS and the Mind-Body Brain-Gut connection.”

^*^Age is expressed in years and presented as mean (standard deviation).

Abbreviations in interventions column are explained directly under intervention; UK: United Kingdom; IBS: irritable bowel syndrome; CIS: the clinical interview schedule; *N*: number of patients; NR: not reported; USA: United States of America; GI: gastrointestinal; CBT: cognitive behavioral therapy; CBG: cognitive-behavioral group therapy; IBD: inflammatory bowel disease; SSRI: selective serotonin reuptake inhibitor; SNRI: serotonin-norepinephrine reuptake inhibitor; TCA: tricyclic antidepressant.

**Table 3:** Subgroup analysis.

Subgroup	SMD	Lower limit	Upper limit	Interaction test
**Risk of bias: high versus low**				
Outcome: IBS composite symptoms severity scales				
High	−0.56	−1.07	−0.04	*P* value = 0.79
Low	−0.64	−0.90	−0.37	

**Intervention: CBT versus other forms of psychotherapy**				
Outcome: IBS composite symptoms severity scales				
CBT	−0.57	−0.86	−0.28	*P* value = 0.56
Other	−0.74	−1.21	−0.26	
Outcome: quality of life scales				
CBT	0.74	0.49	0.99	*P* value = 0.15
Other	0.50	0.28	0.72	

**Control group: received medication versus no medication**				
Outcome: IBS composite symptoms severity scales				
Yes	−0.67	−1.01	−0.33	*P* value = 0.75
No	−0.59	−0.96	−0.23	
Outcome: quality of life scales				
Yes	0.58	0.31	0.85	*P* value = 0.80
No	0.62	0.39	0.86	

SMD: standardized difference in means; IBS: irritable bowel syndrome; CBT: cognitive behavioral therapy.

## Appendix

### Actual Search Strategies

*(1) Ovid*

*Database(s)*. Embase 1988 to 2014 Week 05, Ovid MEDLINE(R) In-Process & Other Non-Indexed Citations and Ovid MEDLINE(R) 1946 to Present, PsycINFO 1967 to January Week 3 2014, EBM Reviews-Cochrane Central Register of Controlled Trials December 2013, EBM Reviews-Cochrane Database of Systematic Reviews 2005 to December 2013.

*Search Strategy* (#, Searches, Results)

1. exp Irritable Bowel Syndrome/, 19426
2. exp Irritable Colon/, 18648
3. exp Colonic Diseases, Functional/, 23156
4. (“Irritable bowel^*^” or “mucous colitis” or “irritable colon^*^” or “functional colonic disease^*^” or colonospasm^*^ or “colon spasm^*^” or “mucomembraneous colitis” or “mucomembranous colitis” or “spastic colon^*^” or “spastic colitis” or “unstable colon^*^” or “mucous colitides” or “colonic spasm^*^”). mp., 27651
5. or/1–4, 29503
6. exp Psychotherapy/, 464651
7. exp mind body therapy/, 76870
8. exp mind-body therapies/, 76826
9. exp Mindfulness/, 2940
10. exp meditation/, 7934
11. exp yoga/, 5954
12. exp stress management/, 6155
13. exp relaxation therapy/, 18867
14. exp relaxation/, 31982
15. exp hypnosis/, 25313
16. exp anxiety management/, 385
17. ((conditioning or psyc^*^ or cognit^*^ or behavior^*^ or stress or anxiety or breath^*^ or “insight-oriented talk” or “insight-oriented verbal” or “acceptance and commitment”) adj3 (therap^*^ or training or treat^*^ or technique^*^ or procedure^*^ or manag^*^ or modifi^*^ or modify or technic or technics or rehab^*^ or restructur^*^)). mp. [mp = ti, ab, sh, hw, tn, ot, dm, mf, dv, kw, nm, kf, px, rx, ui, tc, id, tm, tx, ct], 736491
18. ((stress adj3 reduc^*^) or relaxation or meditat^*^ or hypnotherap^*^ or hypnos^*^ or biofeedback or “bio-feedback” or mindful^*^ or psychotherap^*^ or logotherap^*^ or psychoanaly^*^ or neurofeedback or “sensory feedback” or suggestion^*^ or “behaviour contracting” or “consciousness raising” or yoga or yogic or “mind-body” or mindbody or imagery). mp., 872931
19. or/6–18, 1552410
20. 5 and 19, 3801
21. exp controlled study/, 4359161
22. exp randomized controlled trial/, 710055
23. ((control$ or randomized or randomised) adj2 (study or studies or trial or trials)). mp. [mp = ti, ab, sh, hw, tn, ot, dm, mf, dv, kw, nm, kf, px, rx, ui, tc, id, tm, tx, ct], 5642060
24. meta analysis/, 127132
25. meta-analys$. mp., 220461
26. exp “systematic review”/, 69727
27. (systematic^*^ adj review$). mp., 172449
28. exp Cohort Studies/, 1569846
29. exp longitudinal study/, 1000695
30. exp retrospective study/, 825412
31. exp prospective study/, 670821
32. exp comparative study/, 2454433
33. exp clinical trial/, 1711899
34. exp cross-sectional study/, 283566
35. crossover procedure/, 39737
36. exp cross-over studies/, 97499
37. multivariate analysis/, 186446
38. ((clinical or comparative or cohort or longitudinal or retrospective or prospective or concurrent or “cross-sectional” or crossover or “cross-over”) adj (study or studies or survey or surveys or analysis or analyses or trial or trials)). mp., 7311926
39. (“crossover procedure” or “cross-over procedure” or “multivariate analys^*^”). mp. [mp = ti, ab, sh, hw, tn, ot, dm, mf, dv, kw, nm, kf, px, rx, ui, tc, id, tm, tx, ct], 394850
40. or/21–39, 11388240
41. 20 and 40, 1798
42. from 20 keep 2161–3109, 949
43. limit 42 to (clinical trial, all or clinical trial, phase i or clinical trial, phase ii or clinical trial, phase iii or clinical trial, phase iv or clinical trial or comparative study or controlled clinical trial or evaluation studies or meta analysis or multicenter study or randomized controlled trial or systematic reviews) [Limit not valid in Embase, PsycINFO, CCTR, CDSR; records were retained], 253
44. 41 or 43, 1816
45. limit 44 to (book or book series or editorial or erratum or letter or note or addresses or autobiography or bibliography or biography or comment or dictionary or directory or interactive tutorial or interview or lectures or legal cases or legislation or news or newspaper article or overall or patient education handout or periodical index or portraits or published erratum or video-audio media or webcasts) [Limit not valid in Embase, Ovid MEDLINE(R), Ovid MEDLINE(R) In-Process, PsycINFO, CCTR, CDSR; records were retained], 131
46. 44 not 45, 1685
47. from 20 keep 3567–3801, 235
48. 46 or 47, 1798
49. 48 not (exp animals/ not exp humans/), 1626
50. from 48 keep 1741–1798, 58
51. 49 or 50, 1684
52. limit 51 to yr = “1966–Current”, 1684
53. remove duplicates from 52, 1219

*(2) Scopus*

1. TITLE-ABS-KEY (“Irritable bowel^*^” or “mucous colitis” or “irritable colon^*^” or “functional colonic disease^*^” or colonospasm^*^ or “colon spasm^*^” or “mucomembraneous colitis” or “mucomembranous colitis” or “spastic colon^*^” or “spastic colitis” or “unstable colon^*^” or “mucous colitides” or “colonic spasm^*^”)
2. TITLE-ABS-KEY ((conditioning W/3 therap^*^) or (conditioning W/3 training) or (conditioning W/3 treat^*^) or (conditioning W/3 technique^*^) or (conditioning W/3 procedure^*^) or (conditioning W/3 manag^*^) or (conditioning W/3 modifi^*^) or (conditioning W/3 modify) or (conditioning W/3 technic) or (conditioning W/3 technics) or (conditioning W/3 rehab^*^) or (conditioning W/3 restructur^*^) or (psyc^*^ W/3 therap^*^) or (psyc^*^ W/3 training) or (psyc^*^ W/3 treat^*^) or (psyc^*^ W/3 technique^*^) or (psyc^*^ W/3 procedure^*^) or (psyc^*^ W/3 manag^*^) or (psyc^*^ W/3 modifi^*^) or (psyc^*^ W/3 modify) or (psyc^*^ W/3 technic) or (psyc^*^ W/3 technics) or (psyc^*^ W/3 rehab^*^) or (psyc^*^ W/3 restructur^*^) or (cognit^*^ W/3 therap^*^) or (cognit^*^ W/3 training) or (cognit^*^ W/3 treat^*^) or (cognit^*^ W/3 technique^*^) or (cognit^*^ W/3 procedure^*^) or (cognit^*^ W/3 manag^*^) or (cognit^*^ W/3 modifi^*^) or (cognit^*^ W/3 modify) or (cognit^*^ W/3 technic) or (cognit^*^ W/3 technics) or (cognit^*^ W/3 rehab^*^) or (cognit^*^ W/3 restructur^*^) or (behavior^*^ W/3 therap^*^) or (behavior^*^ W/3 training) or (behavior^*^ W/3 treat^*^) or (behavior^*^ W/3 technique^*^) or (behavior^*^ W/3 procedure^*^) or (behavior^*^ W/3 manag^*^) or (behavior^*^ W/3 modifi^*^) or (behavior^*^ W/3 modify) or (behavior^*^ W/3 technic) or (behavior^*^ W/3 technics) or (behavior^*^ W/3 rehab^*^) or (behavior^*^ W/3 restructur^*^) or (stress W/3 therap^*^) or (stress W/3 training) or (stress W/3 treat^*^) or (stress W/3 technique^*^) or (stress W/3 procedure^*^) or (stress W/3 manag^*^) or (stress W/3 modifi^*^) or (stress W/3 modify) or (stress W/3 technic) or (stress W/3 technics) or (stress W/3 rehab^*^) or (stress W/3 restructur^*^) or (anxiety W/3 therap^*^) or (anxiety W/3 training) or (anxiety W/3 treat^*^) or (anxiety W/3 technique^*^) or (anxiety W/3 procedure^*^) or (anxiety W/3 manag^*^) or (anxiety W/3 modifi^*^) or (anxiety W/3 modify) or (anxiety W/3 technic) or (anxiety W/3 technics) or (anxiety W/3 rehab^*^) or (anxiety W/3 restructur^*^) or (breath^*^ W/3 therap^*^) or (breath^*^ W/3 training) or (breath^*^ W/3 treat^*^) or (breath^*^ W/3 technique^*^) or (breath^*^ W/3 procedure^*^) or (breath^*^ W/3 manag^*^) or (breath^*^ W/3 modifi^*^) or (breath^*^ W/3 modify) or (breath^*^ W/3 technic) or (breath^*^ W/3 technics) or (breath^*^ W/3 rehab^*^) or (breath^*^ W/3 restructur^*^) or ((“insight-oriented talk” or “insight-oriented verbal” or “acceptance and commitment”) W/3 (therap^*^ or training or treat^*^ or technique^*^ or procedure^*^ or manag^*^ or modifi^*^ or modify or technic or technics or rehab^*^ or restructur^*^)))
3. TITLE-ABS-KEY ((stress W/3 reduc^*^) or relaxation or meditat^*^ or hypnotherap^*^ or hypnos^*^ or biofeedback or “bio-feedback" or mindful^*^ or psychotherap^*^ or logotherap^*^ or psychoanaly^*^ or neurofeedback or “sensory feedback” or suggestion^*^ or “behaviour contracting” or “consciousness raising” or yoga or yogic or “mind-body” or mindbody or imagery)
4. TITLE-ABS-KEY ((meta W/1 analys^*^) OR (systematic^*^ W/2 review^*^) OR (control^*^ W/2 stud^*^) OR (control^*^ W/2 trial^*^) OR (randomized^*^ W/2 stud^*^) OR (randomized^*^ W/2 trial^*^) OR (randomised W/2 stud^*^) OR (randomised W/2 trial^*^) or “comparative stud^*^” OR “comparative survey^*^” OR “comparative analys^*^” OR “cohort stud^*^” OR “cohort survey^*^” OR “cohort analys^*^” OR “longitudinal stud^*^” OR “longitudinal survey^*^” OR “longitudinal analys^*^” OR “retrospective stud^*^” OR “retrospective survey^*^” OR “retrospective analys^*^” or “prospective stud^*^” OR “prospective survey^*^” OR “prospective analys^*^” or “concurrent stud^*^” OR “concurrent survey^*^” OR “concurrent analys^*^” or “clinical stud^*^” OR “clinical trial^*^” or “cross-sectional stud^*^” or “cross-sectional analys^*^” or “cross-over stud^*^” or “cross-over analys^*^” or “cross-over procedure” or “crossover stud^*^” or “crossover analys^*^” or “crossover procedure” or “multivariate analys^*^”)
5. PUBYEAR > 1965
6. 1 and (2 or 3) and 4 and 5
7. PMID(0^*^) OR PMID(1^*^) OR PMID(2^*^) OR PMID(3^*^) OR PMID(4^*^) OR PMID(5^*^) OR PMID(6^*^) OR PMID(7^*^) OR PMID(8^*^) OR PMID(9^*^)
8. 6 and not 7
9. DOCTYPE(le) OR DOCTYPE(ed) OR DOCTYPE(bk) OR DOCTYPE(er) OR DOCTYPE(no) OR DOCTYPE(sh)
10. 8 and not 9.
